# Genetic analysis of DAF-18/PTEN missense mutants for the ability to maintain quiescence of the somatic gonad and germ line in *Caenorhabditis elegans* dauer larvae

**DOI:** 10.1093/g3journal/jkac093

**Published:** 2022-04-22

**Authors:** Julia Wittes, Iva Greenwald

**Affiliations:** Department of Biological Sciences, Columbia University, New York, NY 10027, USA

**Keywords:** DAF-18, PTEN, dauer, *Caenorhabditis elegans*, quiescence, gonad

## Abstract

The mammalian tumor suppressor PTEN has well-established lipid phosphatase and protein phosphatase activities. DAF-18, the *Caenorhabditis elegans* ortholog of PTEN, has a high degree of conservation in the catalytic domain, and human PTEN complements a null allele of *daf-18*, suggesting conserved protein function. Insights gleaned from studies of mammalian PTEN have been applied to studies of DAF-18 in *C. elegans*, including predicted enzymatic properties of mutants. Here, we characterize DAF-18 missense mutants previously treated as selectively disrupting either protein or lipid phosphatase activity in genetic assays to connect distinct phenotypes to specific enzymatic activities of DAF-18/PTEN. We analyze the ability of these mutants to maintain quiescence of the somatic gonad and germ line in dauer larvae, a state of diapause during which development is suspended. We show that transgenes expressing either the putative lipid phosphatase-deficient or putative protein phosphatase-deficient form fail to complement a *daf-18* null allele, and that the corresponding homozygous endogenous missense mutant alleles fail to maintain developmental quiescence. We also show that the endogenous *daf-18* missense alleles fail to complement each other, suggesting that one or both of the missense forms are not activity-selective. Furthermore, homozygous *daf-18* missense mutants have a more severe phenotype than a *daf-18* null mutant, suggesting the presence of functionally compromised mutant DAF-18 is more deleterious than the absence of DAF-18. We discuss how these genetic properties complicate the interpretation of genetic assays to associate specific enzymatic activities with specific phenotypes.

## Introduction 

PTEN is a dual-specificity phosphatase that regulates many different cellular processes, and abnormal *PTEN* activity is associated with cancer, autism, metabolic disease, and aging (reviewed in Ortega-Molina and Serrano 2013; Worby and Dixon 2014). PTEN has 2 enzymatic activities: a lipid phosphatase activity and a protein phosphatase activity (Myers *et al.* 1997, 1998; Furnari *et al.* 1998; Maehama and Dixon 1998; Stambolic *et al.* 1998). The *Caenorhabditis* *elegans* PTEN ortholog, DAF-18, also has both activities, and its phosphatase domain shares a high degree of sequence conservation with its human ortholog (Ogg and Ruvkun 1998; Gil *et al.* 1999; Rouault *et al.* 1999; Solari *et al.* 2005; Brisbin *et al.* 2009). Furthermore, transgenic human PTEN can rescue phenotypes caused by the absence of *daf-18* in *C. elegans* (Solari *et al.* 2005; McDiarmid *et al.* 2018; Post *et al.* 2020), suggesting there is also substantial functional conservation. Thus, insights from biochemical and structural studies of mammalian PTEN have been applied to studies of DAF-18 in *C. elegans*.

These insights have included the extrapolation of the effect of missense mutations characterized in mammalian PTEN to DAF-18. In particular, missense mutations that preferentially disrupt lipid or protein phosphatase activity have offered the possibility of connecting specific mutant phenotypes caused by loss of *daf-18* activity to specific enzymatic activities of DAF-18/PTEN. Thus, transgenes that express DAF-18 with missense mutations at equivalent amino acids to human PTEN have been assayed for the ability to rescue *daf-18* null mutants for distinct phenotypes (Solari *et al.* 2005; Nakdimon *et al.* 2012; Zheng *et al.* 2018).

PTEN^G129E^ has little remaining lipid phosphatase activity, but retains significant protein phosphatase activity (Myers *et al.* 1997, 1998; Furnari *et al.* 1998; Ramaswamy *et al.* 1999; Han *et al.* 2000). The analogous mutant form, DAF-18(G174E), was unable to rescue a *daf-18* null mutant in assays of lifespan or dauer entry (Solari *et al.* 2005), or to restore Q neuroblast quiescence during L1 arrest (Zheng *et al.* 2018), and these findings were interpreted as evidence that lipid phosphatase activity is essential to these processes. DAF-18(G174E) only partially restored inhibition of Ras pathway activity during vulval induction, interpreted as indicating that this interaction may be partially independent of lipid phosphatase activity (Nakdimon *et al.* 2012).

PTEN^Y138L^ lacks protein phosphatase activity but appears to retain most lipid phosphatase activity, and is commonly used in mammalian studies; however, the affected residue is not conserved in *C. elegans* (Davidson *et al.* 2010; Tibarewal *et al.* 2012; Berglund *et al.* 2013). Instead, DAF-18(D137A), a mutant form equivalent to PTEN^D92A^ (Tamura *et al.* 1998), which binds to protein substrates but fails to release them, was treated as selectively protein phosphatase-deficient for the purposes of comparison with DAF-18(G174E) (Zheng *et al.* 2018). The ability of DAF-18(D137A) but not DAF-18(G174E) to restore Q neuroblast quiescence during L1 arrest was taken as support for the inference that lipid phosphatase activity was important, and that the different mutant forms preferentially affect different phosphatase activities (Zheng *et al.* 2018).

Here, we describe our analysis of these DAF-18 mutants for regulating quiescence of the somatic gonad and germ line in dauer diapause, a state of reversible developmental arrest that occurs in response to unfavorable environmental conditions (Murphy and Hu 2013; Karp 2018; Baugh and Hu 2020). In favorable conditions, *C. elegans* develops continuously through 4 larval stages (L1–L4) separated by molts, and reaches adulthood in about 3 days. However, if L1 larvae are grown under unfavorable conditions, they molt into second stage “L2d” larvae; if conditions do not improve, the L2d larvae molt into dauer larvae, which can endure for many months.

**Fig. 1.**
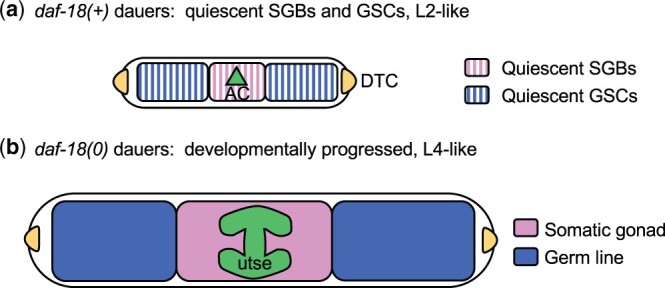
Loss of somatic gonad and germline quiescence in *daf-18(0)* dauers. Gonadogenesis proceeds in 2 phases during continuous development. In the first phase, which ends in L2, the somatic gonad primordium forms; it contains 9 SGBs, 2 differentiated DTCs, and the differentiated AC. It also contains GSCs. Gonadogenesis appears to be suspended at approximately this point in dauer larvae. In the second phase, which begins in L3, the gonad arms extend longitudinally, the SGBs divide and ultimately give rise to descendants, including the uterine seam syncytial cell (utse) formed from the fusion of certain SGB descendants and the AC. The GSCs continue to proliferate; the first meioses begin in the L4 stage, giving rise to sperm. a) Schematic of *daf-18(+)* dauer gonadal anatomy. During dauer, the SGBs and GSCs enter a state of prolonged quiescence. b) Schematic of *daf-18(0)* dauer somatic gonadal anatomy. SGBs and GSCs do not maintain quiescence, and progress developmentally as in continuous development. They generally display morphological characteristics normally associated with the L4 stage of larval development, such as formation of the utse in the somatic gonad and an expanded germ line. In addition, some *daf-18(0)* dauers produce sperm (not diagrammed).

One consequence of dauer entry is the suspension of gonadogenesis. In dauer larvae, somatic gonad blast cells (SGBs) and germline stem cells (GSCs) are maintained in a state of quiescence (Masse *et al.* 2005; Narbonne and Roy 2006; Tenen and Greenwald 2019; Fig. 1a). Loss of *daf-18* in the somatic gonad leads to loss of quiescence of both the SGBs and GSCs (Fig. 1b); expression of DAF-18 in the somatic gonad is able to rescue the progression defect of both populations of cells. It was proposed that DAF-18*/*PTEN in dauer larvae promotes the production or activity of one or more “pro-quiescence” signals in the somatic gonad that maintain quiescence of SGBs and GSCs (Tenen and Greenwald 2019).

In this study, we wanted to gain insight into the identity of the hypothesized pro-quiescence signal(s) by asking if expression of DAF-18 mutant proteins presumed to be selectively defective in lipid [DAF-18(G174E)] or protein [DAF-18(D137A)] phosphatase activity differed in their ability to restore SGB and GSC quiescence in a *daf-18* null mutant background. However, as described herein, our analysis of transgenes and endogenous alleles suggests that these mutations may not be sufficiently activity-selective to allow for such a distinction, with a particular concern about the specificity of DAF-18(D137A) for selective reduction of the protein phosphatase activity. A similar conclusion was reached in a companion study by Chen *et al.* (2022). Together, the 2 studies provide a cautionary tale against comparing these alleles for dissecting the contribution of different enzymatic activities of DAF-18 in *C. elegans* development. In addition, our analysis suggests that the presence of functionally compromised mutant DAF-18 is more deleterious than the absence of DAF-18, a finding with implications for other genetic experiments, such as interpreting results from rescue experiments that rely on multicopy array transgenes.

## Materials and methods

### Strains, alleles, and transgenes

*Caenorhabditis* *elegans* were maintained on NGM plates seeded with OP50 *E. coli*. Strain names and full genotypes used in this study are compiled in Supplementary Table 1. The temperature-sensitive dauer-constitutive mutation *daf-7(e1372)* (Riddle *et al.* 1981) was included in all strains. Strains were maintained at 15°C; embryos or L1 larvae were shifted to the restrictive temperature of 25°C to induce dauer entry.

The *daf-18*(*ok480)* allele contains a 956 bp deletion that removes most of exon 4 and all of exon 5, and ends in intron 5, and is generally considered to be a null allele (Kennedy *et al.* 2013; Liu *et al.* 2014; McDiarmid *et al.* 2018; Shi *et al.* 2020; Fry *et al.* 2021). Two additional alleles, *daf-18{syb1615[daf-18(D137A)]}* and *daf-18{syb1618[daf-18(G174E)]}*, were a gift from Ryan Baugh and Jingxian Chen (see accompanying paper by Chen *et al.* 2022). We note that *daf-18(syb1615)* and *daf-18(syb1618)* prevent dauer formation in a *daf-7(+)* background based on lack of SDS-resistant dauers on starved plates (Cassada and Russell 1975; Karp 2018). This dauer-defective phenotype was quantified by Chen *et al.* using suppression of dauer formation by *daf-2(RNAi)* (see Chen *et al.* 2022).

The strain VC4510 containing *nog-1(gk5581[loxP + myo-2p::GFP::unc-54 3’UTR + rps-27p::neoR::unc-54 3’UTR + loxP]) IV* (Au *et al.* 2019) was obtained from the CGC. The *nog-1(gk5581)* allele was used as a genetic balancer for *daf-18* due to the close proximity of *nog-1* and *daf-18*.

The following transgenes were used:

*arIs51[cdh-3p::GFP] IV* (Karp and Greenwald 2003) marks the anchor cell (AC) following its specification in L2 or L2d. In stage L4 and in *daf-18(0)* dauer larvae, *arIs51* expression spreads to encompass multiple nuclei, reflecting formation of the uterine seam syncytial cell (utse) from the fusion of the AC to other uterine cells (Newman *et al.* 1995; Tenen and Greenwald 2019).

*arSi40[mex-5p::2xmTagBFP2::his-11::tbb-2 3’UTR] I* is an insertion in the *ttTi4348* site on LGI, generated by Claire de la Cova (unpublished). This transgene is expressed in all germ cells, including GSCs, sperm, and oocytes.

### Production of single copy DAF-18 rescue transgenes

Transgenes were assembled in the pWZ111 vector, which contains a self-excising cassette (SEC) (Dickinson *et al.* 2015) and homology arms for the *ttTi4348* site on linkage group I (LGI); this vector was used to generate single-copy transgenes at a consistent site in the genome. To make pJSW56(*ckb-3p::daf-18cDNA::gfp::unc-54 3’UTR*), pWZ111 cut with NotI and AvrII was used as the plasmid backbone. *ckb-3p::S::daf-18cDNA* was amplified from pJSW37. “S” stands for synthetic intron; this cassette includes a Kozak sequence, and was included to enhance DAF-18 expression (Kozak 1986; Fire *et al.* 1990; Tenen and Greenwald 2019). The *gfp::unc-54 3′UTR* was amplified from pCT63; this *gfp* sequence is worm codon-optimized and includes 3 synthetic introns (Dickinson *et al.* 2015). pJSW56 was then assembled by Gibson cloning (HiFi reagent, NEB Inc., MA). To generate pJSW59*[ckb-3p::daf-18(D137A)cDNA::gfp::unc-54 3′UTR]*, pJSW60*[ckb-3p::daf-18(C169S)cDNA::gfp::unc-54 3′UTR]*, and pJSW61*[ckb-3p::daf-18(G174E)cDNA::gfp::unc-54 3′UTR]*, pWZ111 cut with NotI and AvrII was used as the vector backbone, and mutations were introduced into pJSW56 by Gibson assembly using primers incorporating the desired mutations. To produce D137A, codon 137 (GAT) was mutated to GCT. To produce C169S, codon 169 (TGT) was mutated to TCA. To produce G174E, codon 174 (GGC) was mutated to GAA. All plasmids were verified by sequencing.

Transgenes were generated by microinjection into the germ line of wildtype Bristol N2 hermaphrodites by the approach of Pani and Goldstein (2018). Prior to injection, plasmids were purified with the Purelink miniprep kit (ThermoFisher). DAF-18 rescuing plasmids were injected at 50 ng/µl. Injection mixes included the plasmid pAP082, which expresses Cas9 in the germ line and an sgRNA targeting *ttTi4348* on LG I (50 ng/µl). The coinjection markers pGH8 (10 ng/µl) and pCFJ90 (2.5 ng/µl), were also included in injection mixes. Worms bearing single copy insertions were selected using the dominant roller and hygromycin resistance genes present in the SEC. The SEC was then excised by heat shock. We note that *ckb-3p::daf-18::gfp::unc-54 3′UTR* expression could not be detected in the somatic gonad in dauer larvae. DAF-18::GFP was also not detectable in early L1, when *ckb-3* promoter expression is strongest.

Single copy rescue transgenes generated for this study are:

*arSi97[ckb-3p::daf-18::gfp::unc-54 3′* *UTR]*, from pJSW56

*arSi120[ckb-3p::daf-18(D137A)::gfp::unc-54 3′* *UTR]*, from pJSW59

*arSi108[ckb-3p::daf-18(C169S)::gfp::unc-54 3′* *UTR]*, from pJSW60

*arSi112[ckb-3p::daf-18(G174E)::gfp::unc-54 3′* *UTR]*, from pJSW61

### Preparation and imaging of dauer larvae

Partially synchronized populations of worms were obtained for imaging using either of 2 methods. For *daf-18(0)* rescue experiments, eggs were isolated by hypochlorite treatment (Stiernagle 2006) and shifted to 25°C to induce dauer formation. Animals were imaged at 72 h post-egg prep (PEP), at which point we estimate they have been in dauer for approximately 31–45 h. For all other experiments, embryos and L1s were isolated by a 24 h egg lay at 15°C and then shifted to 25°C. Dauers were imaged 48 h after the shift to 25°C, and we estimate worms have been in dauer for up to 29 h at this timepoint.

Dauers were isolated at the desired timepoint by treating worms with 1% SDS (Cassada and Russell 1975; Karp 2018) for 10 min. Dauers were then mounted on 4% agarose pads and immobilized with 2 µl of 10 mM levamisole in M9 and imaged. Only hermaphrodite dauer larvae were scored. Cross progeny were identified as such by the presence of the germline marker. Hermaphrodite dauers were identified by the presence of 2 gonad arms, as males do not have 2 arms. Furthermore, the germ line is segregated into the 2 gonad arms in the hermaphrodite dauer and clusters at the gonad posterior in the male. In 4/44 *daf-18* trans-heterozygous dauers, the gonad morphology was sufficiently aberrant that they could not be confidently sexed based on these criteria and so they were excluded from further analysis.

For rescue experiments, imaging and scoring was performed using a Zeiss Axio Imager D1 microscope with a Zeiss AxioCam MRm camera and 40x Plan-Neo and 63x Plan-Apo objectives. All other imaging was performed using a Cell Observer SD spinning disc confocal microscope (Carl Zeiss) with a 40X, 1.4 NA objective. Five micrometers Z-slices were acquired in all Z-planes containing detectable germ cell nuclei, marked by *arSi40[mex-5p::2xmTagBFP2::his-11::tbb-2 3′UTR].* Confocal images were acquired with a Hamamatsu Orca flash 4.0 LT+ CMOS camera.

### Quantifying somatic gonad progression using *arIs51*

Dauer larvae with detectable *arIs51* expression in a swath of multiple cells were scored as “progressed” (Tenen and Greenwald 2019), and dauers with expression in 1 cell (or, very occasionally, in 0 cells or 2 cells both appearing to be ACs) were scored as “not progressed”. The percentage of animals displaying evidence of somatic gonad progression for a given genotype was computed, and two-tailed Fisher’s exact tests were performed using R to judge whether the penetrance of somatic gonad progression differed among genotypes. Plots were made with GraphPad Prism 9 software.

### Quantifying germline progression using *arSi40*

GSCs were manually counted in ImageJ. Sperm were not included in the GSC count. For some animals, GSCs could not be accurately counted because the *arSi40* marker was too dim, because of movement during imaging, or because of issues with mounting, such as the presence of a bubble close to a worm. Brown-Forsythe and Welch ANOVA tests with Dunnett’s T3 test for multiple comparisons were used to analyze GSC count data; plots were generated in Prism.

## Results

At hatching, the *C. elegans* gonad consists of 2 somatic gonad progenitor cells, Z1 and Z4, and 2 germline progenitor cells, Z2 and Z3. During the first phase of gonadogenesis, which is completed by the end of the L2 stage, Z1 and Z4 generate a 12-cell somatic gonad primordium, which includes 9 SGBs and 3 terminally differentiated cells: the 2 distal tip cells (DTCs) and the AC (Kimble and Hirsh 1979). The second phase of gonadogenesis, which occurs in the L3 and L4 stages during continuous development, is associated with landmark developmental events, including the production of a multinucleate syncytium called the utse, generated by fusion of the AC and descendants of the ventral uterine precursor cells (VUs) in the L4 stage (Kimble 1981; Newman *et al.* 1995), and the extension and reflexion of the gonad arms led by the DTCs (Kimble 1981; Kimble and White 1981). The germline progenitors Z2 and Z3 undergo mitosis in L1 and generate the germline stem cells (GSCs) (Hubbard and Schedl 2019). The DTCs regulate division of the GSCs and the timing of meiotic entry; the GSCs proliferate mitotically until the L4 stage, when the GSCs furthest from the DTCs enter meiosis and produce sperm. In adult hermaphrodites, the GSCs produce gametes that differentiate as oocytes.

### *daf-18* missense mutants lack quiescence-promoting activity in the *daf-18(0)* dauer gonad

The decision to enter and exit dauer involves integrating information about population density, temperature, and food availability. Food availability and population density are sensed by the insulin/IGF-1 signaling (IIS) and transforming growth factor Beta (TGF-β) signaling pathways (reviewed in Murphy and Hu 2013; Baugh and Hu 2020). In the presence of food, IIS is active, leading to activation of PI3K and the production of PIP3 from PIP2; in the absence of food, when IIS is inactive, low PIP3 levels favor dauer entry. DAF-18/PTEN opposes IIS activity by dephosphorylating PIP3. Thus, in mutants lacking DAF-18/PTEN, high PIP3 levels are able to accumulate even when the food signal is absent, opposing dauer entry, and *daf-18* single mutants are dauer-defective. However, dauers lacking *daf-18* can be made by compromising *daf-7/*TGF-β signaling, a parallel input into the decision to enter dauer (Vowels and Thomas 1992; Larsen *et al.* 1995; Narbonne and Roy 2006). *daf-7(e1372ts)* produces obligate dauer entry in young worms (embryos or L1s) raised at 25°C, and all *daf-18(+)* and *daf-18* mutant dauers in this study also carry the *daf-7(e1372*) allele.

Dauer diapause induced by unfavorable environmental conditions or by a *daf-7* mutation is associated with the suspension of gonadogenesis after the first phase and the cessation of GSC mitosis (Fig. 1a). In *daf-18(+)* dauer larvae, the SGBs and germline stem cells (GSCs) are quiescent; in *daf-18(0)* dauers, they are not, such that the SGBs continue to progress developmentally and generate differentiated descendants, and the GSCs proliferate mitotically (Narbonne and Roy 2006; Tenen and Greenwald 2019; Fig. 1b). A single-copy insertion transgene that expresses DAF-18(+) in the somatic gonad cells under the control of the *ckb-3p* regulatory sequence (Kroetz and Zarkower 2015) is sufficient to restore quiescence to both SGBs and GSCs (Tenen and Greenwald 2019).

**Fig. 2.**
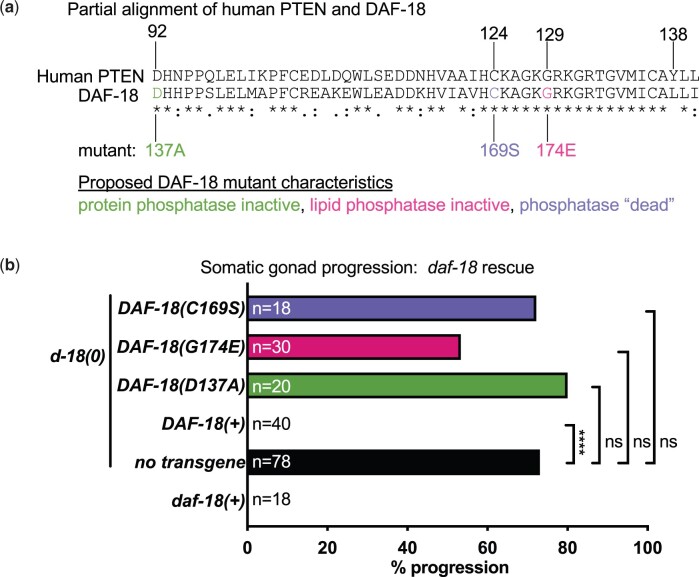
Developmental quiescence of the *daf-18(0)* dauer gonad is not restored by rescue constructs with mutant *daf-18* alleles. a) Partial alignment of human PTEN and *C. elegans* DAF-18. Residues referenced in this study are indicated. (*) Identical, (:) conservative, (.) semi-conservative. A complete sequence alignment of DAF-18 and human PTEN can be found in McDiarmid *et al.* (2018). In addition to the DAF-18(G174E) and DAF-18(D137A) mutants, we also assayed DAF-18(C169S), which is thought to be equivalent to PTEN^C124S^ and to lack both phosphatase activities (Barford *et al.* 1994; Myers *et al.* 1997, 1998; Maehama and Dixon 1998; Lee *et al.* 1999); it may also have “substrate-trapping” properties i.e. binding to but not releasing substrates (Flint *et al.* 1997; Myers *et al.* 1997, 1998; Maehama and Dixon 1998; Ramaswamy *et al.* 1999; Qi *et al.* 2020). b) Quantification of somatic gonad progression in *daf-18(0)* dauers expressing single-copy DAF-18 transgenes, scored with *arIs51[cdh-3p::gfp]*. Expression of wildtype DAF-18 in the somatic gonad fully restored somatic gonad quiescence. None of the 3 DAF-18 mutants significantly rescued the progression defect. ****P < 0.0001 by two-tailed Fisher’s exact test, ns = no significant difference. Full genotypes can be found in Supplementary Table 1.

As a first step toward identifying the hypothesized pro-quiescence signal or signals originating in the somatic gonad and acting on the SGBs and GSCs (Tenen and Greenwald 2019), we wanted to identify the relevant phosphatase activity or activities of DAF-18 for maintaining SGB and GSC quiescence using missense mutations reported to be selectively deficient in the protein phosphatase (D137A) or lipid phosphatase (G147E) activity of DAF-18, or in both phosphatase activities (C169S) (see Introduction and Fig. 2). We generated single-copy insertion transgenes expressing DAF-18 missense mutant proteins tagged with GFP at the carboxy terminus under control of the somatic gonad-specific *ckb-3* promoter (Kroetz and Zarkower 2015) at a common site on chromosome I to provide a uniform genomic context (*Materials and Methods*). *daf-18(0)* dauer larvae carrying these transgenes were examined 72 h post egg prep (Fig. 2b), at which point we estimate they have been in dauer diapause for 31–45 h (Tenen and Greenwald 2019).

To assess the maintenance of SGB quiescence, we used the fluorescent reporter *arIs51[cdh-3p::gfp]* (Karp and Greenwald 2003). During continuous development, GFP fluorescence is restricted to the AC in the L3 stage, but in the L4 stage, expression expands throughout the utse syncytium formed by fusion of descendants of 3 SGBs (the ventral uterine precursor cells) and the AC (Newman *et al.* 1995). In *daf-18(+)* dauers, *arIs51* expression in the gonad is restricted to the AC, but in most *daf-18(0)* dauers, expression expands to a utse-like structure (Fig. 1), reflecting developmental progression to the equivalent of the L4 stage (Tenen and Greenwald 2019). Expression of transgenic DAF-18(+)::GFP resulted in full rescue of the somatic gonad progression defect (Fig. 2b), although fluorescence from DAF-18(+)::GFP was not visible in any somatic gonad cells. None of the *daf-18::gfp* mutant transgenes (Fig. 2a) had significant rescuing activity (Fig. 2b). Although we did not quantify GSCs in this experiment, the width of the gonad arm appeared to be expanded, which is indicative of GSC progression in dauer larvae (Hong *et al.* 1998; Narbonne and Roy 2006; Tenen and Greenwald 2019).

The inability of the D137A and G174E mutants to restore SGB quiescence may indicate that both the lipid and protein phosphatase activities are required to maintain quiescence. However, it also seemed possible that the assumptions made about these alleles are not correct, and that they are not as selective as presumed. Using endogenous alleles with the same amino acid changes, the question of selectivity then became the focus of our work.

### *daf-18(D137A)* and* daf-18(G174E)* mutants lose quiescence in the somatic gonad and germ line

**Fig. 3.**
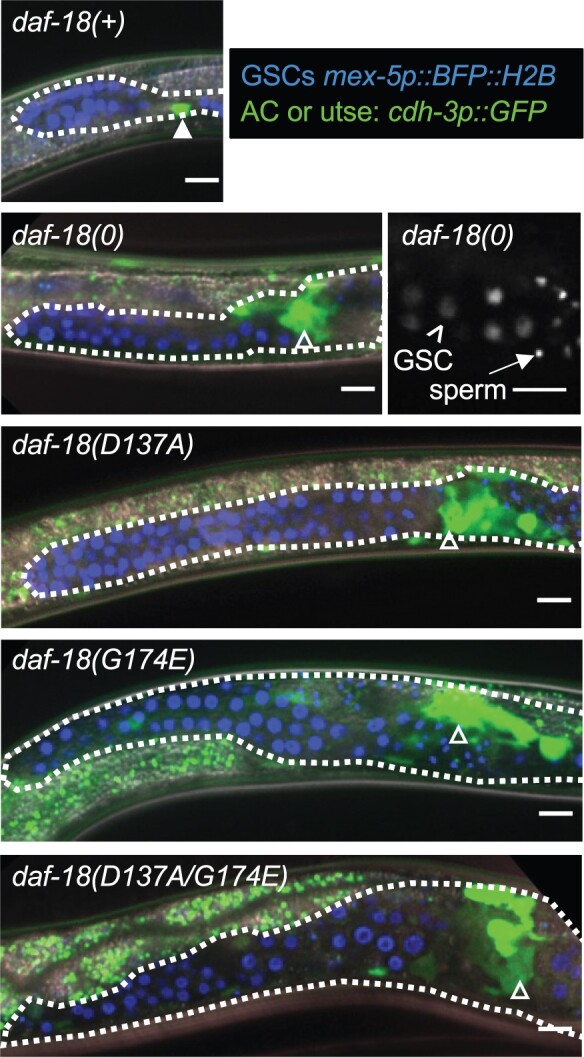
Representative images of the anterior gonad arm for genotypes analyzed in Fig. 4. Full genotypes can be found in Supplementary Table 1. *arIs51[cdh-3p::gfp]* is restricted to the AC (closed arrowhead) in the quiescent gonad of *daf-18(+)* dauers. Expanded GFP expression reflects developmental progression of the somatic gonad and formation of a utse-like structure (open arrowhead) in *daf-18* mutant dauers. For each genotype, the anterior arm is featured and the outline of the gonad is delimited by a white dotted line. Germ cell nuclei are marked with *arSi40[mex-5p::bfp::h2b]*. Sperm were present in the proximal gonad of all *daf-18* larvae shown in this figure. Sperm nuclei are characteristically more compact than GSC nuclei; see grayscale panel depicting sperm and GSC nuclei (marked by *arSi40*) in the proximal gonad of a *daf-18(0)* dauer larva. Scale bars are 10 µm.

**Fig. 4.**
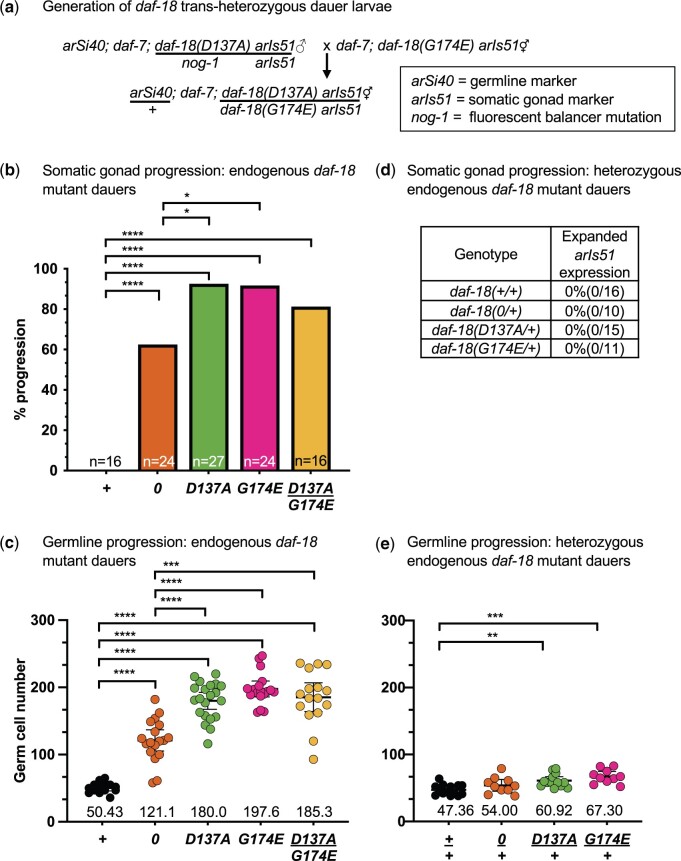
Characterization of somatic gonad and germline progression for endogenous *daf-18* alleles. All genotypes include *daf-7(e1372)* to drive dauer formation and the markers *arIs51[cdh-3p::gfp]* and *arSi40[mex-5p::bfp::h2b]*. Full genotypes can be found in Supplementary Table 1. Only statistically significant differences are shown. a) Mating scheme for generating *daf-18* trans*-*heterozygous dauer larvae. See *Materials and Methods* for marker details. b) Somatic gonad progression. All *daf-18* homozygous mutant genotypes displayed significant developmental progression compared to *daf-18(+)* dauers. Progression occurred with a higher penetrance in *daf-18(G174E)* and *daf-18(D137A)* dauers than in *daf-18(0)* dauers. ****P < 0.0001, *P < 0.05, two-tailed Fisher’s exact test. c) Germline progression. All *daf-18* homozygous mutant genotypes had significantly more GSCs compared to *daf-18(+)* dauers. *daf-18(D137A)*, *daf-18(G174E)* and *daf-18(D137A/G174E)* dauers have significantly more GSCs than *daf-18(0)* or *daf-18(+)* dauers. For each genotype, in-graph numbers are mean GSC numbers. Error bars represent 95% confidence intervals. P-values were computed using Brown-Forsythe and Welch ANOVAs with Dunnett’s T3 test for multiple comparisons. ****P < 0.0001, ***P < 0.001. Sperm were present in some *daf-18* homozygous and trans-heterozygous dauers, which reflects L4-like germline progression. d) Somatic gonad progression was not observed in *daf-18/+* heterozygous dauers. Somatic gonad progression was assessed using *arIs51[cdh-3p::gfp]*. e) Weak semi-dominant germline progression was observed in *daf-18(D137A/+)* and *daf-18(G174E/+)* heterozygous dauers. In-graph numbers, error bars, and statistical testing are as in c. The small effect was only apparent by counting the number of GSCs, as the overall anatomy was indistinguishable from *daf-18(+)* dauers; sperm were never observed. **P < 0.01, ***P < 0.001.

We next examined the phenotypes associated with the endogenous mutations *daf-18(syb1615)*, hereafter denoted as *daf-18(D137A)*, and *daf-18(syb1618)*, denoted as *daf-18(G174E)*, kindly provided by Jingxian Chen and Ryan Baugh (see Chen *et al.* 2022), using *daf-7(e1372*) to drive dauer formation as described above. *daf-18(D137A)* and *daf-18(G174E)* dauers, like *daf-18(0)* dauers, displayed an enlarged gonad, which was readily visible by DIC microscopy (Fig. 3). Furthermore, somatic gonad progression to the formation of the utse was detected in a significantly higher fraction of *daf-18(D137A)* and *daf-18(G174E)* dauers (≥92%) than *daf-18(0)* dauers (63%) (Fig. 4b).

We also quantified germline progression using the nuclear-localized germline marker *arSi40[mex-5p::2xmTagBFP2::his-11::tbb-2 3’UTR]*, which marks GSCs and differentiated germ cells. This analysis revealed that *daf-18(G174E)* and *daf-18(D137A)* dauers have significantly more GSCs than *daf-18(+)* and *daf-18(0)* homozygotes (Fig. 4c). Sperm production normally begins in the L4 stage, and sperm were never detected in *daf-18(+)* dauers (*n* = 0/16); however, sperm were observed in *daf-18(0)* (*n* = 8/24; 33%), *daf-18(D137A)* (*n* = 11/27; 41%) and *daf-18(G174E)* (*n* = 10/24; 42%) dauers (see Fig. 3). The presence of sperm in *daf-18* dauers has not been reported previously, and their presence suggests that the germ line, like the somatic gonad, progresses to an L4-equivalent stage in *daf-18* dauers.

The developmental progression observed for the endogenous *daf-18(G174E)* and *daf-18(D137A)* alleles is consistent with the inability of the equivalent mutant forms to rescue developmental progression in *daf-18(0)* dauers when expressed from transgenes, as described above. Importantly, the higher penetrance of somatic gonad defects and more severe germline defects in *daf-18(G174E)* and *daf-18(D137A)* dauers than *daf-18(0)* suggests that the presence of functionally compromised mutant DAF-18 is more deleterious than the absence of DAF-18.

### *daf-18(D137A)* and *daf-18(G174E)* alleles fail to complement

If the *daf-18(D137A)* and *daf-18(G174E)* alleles were truly selective for different phosphatase activities, we reasoned that they would display intragenic complementation. As a prelude to performing the complementation test, we assessed the *daf-18* null allele for haploinsufficiency and the missense alleles for dominance, properties that would preclude complementation testing.

To assess haploinsufficiency, we examined *daf-18(0/+)* dauers, and found that the *arIs51* marker was always restricted to the AC (Fig. 4d) and the gonads of heterozygous animals were comparable in size and shape to *daf-18(+/+)* dauers. There was also no significant difference in germ cell number between *daf-18(0/+)* dauers and *daf-18(+/+)* dauers (Fig. 4e). These results indicate that *daf-18* is not haploinsufficient.

To assess the dominance of missense mutations, we examined *daf-18* missense mutant heterozygous dauers. The somatic gonad resembled *daf-18(+/+)* dauers, indicating that these alleles are recessive for this phenotype; similarly, the gonad arms were not overtly expanded and sperm were not seen, consistent with the mutations being recessive for loss of germline quiescence and developmental progression. However, the number of GSCs in the *daf-18(D137A/+)* and *daf-18(G174E/+)* heterozygotes is slightly greater than in control *daf-18(+/+)* dauers (Fig. 4e), suggesting that there may be a weak dominant-negative effect of these missense mutations, which may occur during or prior to dauer entry (Narbonne and Roy 2006), even though overt developmental progression is halted in dauer (compare Fig. 4, c and e).

We therefore proceeded with the complementation test, and found that *daf-18(D137A/G174E)* trans-heterozygotes did not display complementation with respect to somatic gonad (Fig. 4b) or GSC (Fig. 4c) quiescence. Furthermore, the number of GSCs in *daf-18(G174E/D137A)* trans-heterozygous dauers and the penetrance of somatic gonad progression (81%) is not statistically different from that of *daf-18(G174E)* and *daf-18(D137A)* single mutants, and sperm are present in trans-heterozygous dauers (*n* = 6/16; 38%). These results suggest that the *daf-18(D137A)* and *daf-18(G174E)* alleles do not display distinct functional separation of lipid and protein phosphatase activities. We cannot distinguish from these data whether one or both alleles are not sufficiently selective to allow for discrimination of different activities genetically, and we describe further considerations below.

## Discussion

In this study, we investigated the ability of DAF-18 missense mutant proteins that had been assumed to selectively lack either protein or lipid phosphatase activities to maintain developmental quiescence of the somatic gonad and/or germ line in dauer larvae. We found that transgenes encoding the DAF-18(D137A) putative protein phosphatase-lacking and DAF-18(G174E) putative lipid phosphatase-lacking forms, as well as the corresponding endogenous missense alleles of *daf-18*, appear unable to maintain quiescence of the somatic gonad or germ line of dauer larvae. A similar finding was made by Chen *et al.* (see accompanying paper) in their studies of the ability of these alleles to promote starvation resistance during L1 arrest.

Taken at face value, our results for the single mutants imply that both the lipid and protein phosphatase activities of DAF-18 are required for maintaining developmental quiescence in dauer larvae. However, we also analyzed the trans-heterozygote *daf-18(D137A)/daf-18(G174E)* and found that the alleles failed to complement for maintaining quiescence of the somatic gonad and germ line in dauer larvae. If both alleles were truly selective, and if both phosphatase activities were indeed required, we would have expected them to complement each other. Chen *et al.* have found that *daf-18(D137A)* and *daf-18(G174E)* also fail to complement for promoting starvation resistance during L1 arrest. Thus, we believe that one or both alleles are not sufficiently selective to allow for discrimination of different activities genetically, and that our findings offer a cautionary tale for their use in associating a specific mutant phenotype caused by loss of *daf-18* activity to a specific enzymatic activity of DAF-18/PTEN.

Revisiting the mammalian literature that directed the mutations used to generate these alleles, there is considerable evidence that PTEN^G129E^ selectively lacks lipid phosphatase activity relative to protein phosphatase activity (Myers *et al.* 1997, 1998; Furnari *et al.* 1998; Ramaswamy *et al.* 1999; Han *et al.* 2000), whereas there is less support for the contention that PTEN^D92A^ selectively lacks protein phosphatase activity. Indeed, there is evidence that PTEN^D92A^ has reduced lipid phosphatase activity (Furnari *et al.* 1998; Lee *et al.* 1999; Ramaswamy *et al.* 1999; Xiao *et al.* 2007; Rodríguez-Escudero *et al.* 2011), so DAF-18(D137A) may be deficient in both phosphatase activities, accounting for the failure of complementation in trans-heterozygotes even if DAF-18(G174E) does selectively lack lipid phosphatase activity.

Our finding that homozygous *daf-18* G174E and D137A mutant dauers have more GSC proliferation than homozygous *daf-18(0)* dauers indicates that some forms of DAF-18 are more deleterious than its complete absence. Additional complexities in using these alleles for genetic analysis can be inferred: for instance, they may provide misleading results in rescue assays, particularly if conventional multicopy arrays are used and a negative result is obtained. Given the sensitivity and ease of quantification of GSC proliferation in dauer larvae, it may be a useful assay for exploring the deleterious effects of other mutations in DAF-18 and human PTEN, given the demonstrated ability to human PTEN to substitute for DAF-18 (Solari *et al.* 2005; McDiarmid *et al.* 2018; Post *et al.* 2020).

## Data availability

The data underlying this article are available in the article and in its Supplementary material online. All strains, plasmids, transgenes, and sequences generated for this study are available upon request.

Supplemental material is available at *G3* online.

## Supplementary Material

jkac093_Supplementary_Data
