# Supplementary material for: Genetic analysis of DAF-18/PTEN missense mutants for the ability to maintain quiescence of the somatic gonad and germ line in Caenorhabditis elegans dauer larvae
Source: G3 (Bethesda). 2022 Apr 22;12(6):jkac093. doi: 10.1093/g3journal/jkac093 (PMC9157151; doi:10.1093/g3journal/jkac093)
Supplement: jkac093_Supplementary_Data [file jkac093_supplementary_data.pdf]

Strain name	Genotype	Source	Figures
GS8052	*daf-7(e1372) III; arIs51[cdh-3p::GFP] IV*	Tenen and Greenwald 2019	2B
GS8024	*daf-7(e1372) III; daf-18(ok480) arIs51 IV*	Tenen and Greenwald 2019	2B
GS9607	*arSi97[ckb-3p::DAF-18-GFP] I; daf-7(e1372) III; daf-18(ok480) arIs51 IV*	This paper	2B
GS9693	*arSi120[ckb-3p::DAF-18(D137A)-GFP] I; daf-7(e1372) III; daf-18(ok480) arIs51 IV*	This paper	2B
GS9663	*arSi108[ckb-3p::DAF-18(C169S)-GFP] I; daf-7(e1372) III; daf-18(ok480) arIs51 IV*	This paper	2B
GS9664	*arSi112[ckb-3p::DAF-18(G174E)-GFP] I; daf-7(e1372) III; daf-18(ok480) arIs51 IV*	This paper	2B
GS9610	*arSi40 I; daf-7(e1372) III; arIs51 IV*	This paper	3, 4B, 4C
GS9611	*arSi40 I; daf-7(e1372) III; daf-18(ok480) arIs51 IV*	This paper	3, 4B, 4C
GS9786	*arSi40 I; daf-7(e1372) III; daf-18(syb1615) arIs51 IV*	This paper	3, 4B, 4C
GS9787	*arSi40 I; daf-7(e1372) III; daf-18(syb1618) arIs51 IV*	This paper	3, 4B, 4C
	arSi40/+; daf-7(e1372) III; daf-18(syb1615) arIs51/daf-18(syb1618) arIs51 IV	This paper	3, 4B, 4C
GS9737	*arSi40 I; daf-7(e1372) III; arIs51 IV cross with males*	This paper	
GS9777	*arSi40 I; daf-7(e1372) III; nog-1(gk5581) arIs51/nog-1(+) arIs51 IV*	This paper	
GS9653	*daf-7(e1372) III; daf-18(syb1615) arIs51 IV*	This paper	
GS9685	*daf-7(e1372) III; daf-18(syb1618) arIs51 IV*	This paper	
	arSi40/+; daf-7(e1372) III; arIs51 IV	This paper	4D, 4E
	arSi40/+; daf-7(e1372) III; daf-18(ok480) arIs51/daf-18(+) arIs51 IV	This paper	4D, 4E
	arSi40/+; daf-7(e1372) III; daf-18(syb1615) arIs51/daf-18(+) arIs51 IV	This paper	4D, 4E
	arSi40/+; daf-7(e1372) III; daf-18(syb1618) arIs51/daf-18(+) arIs51 IV	This paper	4D, 4E

Table S1. Strains and genotypes used in this study. Dauers used for Figures 4D and 4E were cross progeny, produced by crossing GS9737 males to GS9653, GS9685, GS8052 or GS8024 hermaphrodites.
